# The Subcutaneous Injection of Quinine

**Published:** 1902-03-01

**Authors:** 


### THE SUBCUTANEOUS INJECTION OF QUININE.

The superiority of the subcutaneous injection of quinine over its administration by the mouth in certain cases of malaria has long been recognised, although many causes have intervened to prevent the universal adoption of this method. Among these objections may be mentioned the difficulty of dissolving the ordinary sulphate of quinine without adding so much acid as to cause the solution to become very irritating and painful, and, in hot climates, the fear, which seems a very real fear in some places, of causing tetanus. Much would seem to depend upon the preparation employed, the salt usually recommended being the acid hydrochloride, which is very soluble. Dr. Ferguson,[1] of Cheltenham, however, endorses the recommendation of the bi- or acid hydrobromate of quinine, as being far superior to other preparations for this purpose. The examples given by Dr. Ferguson are very striking, half a dozen subcutaneous injections having been sufficient to cure many cases of long standing which had already been subjected to treatment by quinine in other forms without effect. In explaining the superior efficacy of hypodermic injections as compared with administration by the mouth, he refers to the rapid excretion of the drug by way of the kidneys as being the cause of the apparent inactivity of quinine in some cases. The excretion of quinine through the kidneys begins a few minutes after a dose has been given, and it is this fact that explains the superior results from its subcutaneous use. When slowly absorbed through disordered stomach and intestinal membranes it is excreted nearly as fast as it is absorbed and never reaches in the blood a strength sufficient to destroy the malaria plasmodia. On the other hand, when subcutaneously used, its maximum and effective strength is probably reached within half an hour. As to method, Dr. Ferguson injects subcutaneously 3 grains of quinine bi-hydrobromate dissolved in 20 minims of pure warm water. He first injects it under the skin of the upper arm, then under that of the thighs, then under the skin of the abdomen, or at the top of the chest or between the scapulæ. Six injections on alternate days are usually required in a serious case. He keeps a syringe for the purpose, and uses it for nothing else, disinfecting it and the patient's skin with strong carbolic lotion, carefully disinfecting his own hands, and sterilising the needle in the flame of a spirit lamp. The solution of quinine is sterilised when first made, but he has not himself boiled it each time before using, although he now recommends that this should be done. With such precautions, he is convinced that there need be no fear of tetanus, and further he is convinced that there are few or no cases of malarial fever that will resist six subcutaneous injections of 3 grains each of quinine bi-hydrobromate.

[1] Brit. Med. Jour., Feb. 22.

